# Essential elements of physical fitness analysis in male adolescent athletes using machine learning

**DOI:** 10.1371/journal.pone.0298870

**Published:** 2024-04-02

**Authors:** Yun-Hwan Lee, Jisuk Chang, Ji-Eun Lee, Yeon-Sung Jung, Dongheon Lee, Ho-Seong Lee

**Affiliations:** 1 Department of Exercise and Medical Science, Graduate School, Dankook University, Cheonan, Republic of Korea; 2 Institute of Medical-Sports, Dankook University, Cheonan, Republic of Korea; 3 Department of Sports Management, Dankook University, Cheonan, Republic of Korea; 4 The Sport Science Center in Gyeonggi, Seoul, Republic of Korea; 5 Department of Biomedical Engineering, Chungnam National University Hospital, Daejeon, Republic of Korea; 6 Department of Biomedical Engineering, Chungnam National University College of Medicine, Daejeon, Republic of Korea; Università degli Studi di Milano: Universita degli Studi di Milano, ITALY

## Abstract

Physical fitness (PF) includes various factors that significantly impacts athletic performance. Analyzing PF is critical in developing customized training methods for athletes based on the sports in which they compete. Previous approaches to analyzing PF have relied on statistical or machine learning algorithms that focus on predicting athlete injury or performance. In this study, six machine learning algorithms were used to analyze the PF of 1,489 male adolescent athletes across five sports, including track & field, football, baseball, swimming, and badminton. Furthermore, the machine learning models were utilized to analyze the essential elements of PF using feature importance of XGBoost, and SHAP values. As a result, XGBoost represents the highest performance, with an average accuracy of 90.14, an area under the curve of 0.86, and F1-score of 0.87, demonstrating the similarity between the sports. Feature importance of XGBoost, and SHAP value provided a quantitative assessment of the relative importance of PF in sports by comparing two sports within each of the five sports. This analysis is expected to be useful in analyzing the essential PF elements of athletes in various sports and recommending personalized exercise methods accordingly.

## Introduction

In order to improve the physical fitness (PF) and performance of athletes, body composition, muscular strength, muscular endurance, power, agility, and coordination are required [1,2], and it is important to establish a training plan through evaluation and management of PF. Each sport needs particular PF components, for instance, track and field athletes necessitate explosive power, agility, flexibility, muscular endurance, and coordination [3], whereas maximal speed and rapid changes of direction are crucial for football players [4,5]. Baseball players demand substantial muscle strength to achieve quick pitching and batting [6], whereas swimmers require upper body and core strength to execute fast strokes, and badminton players need muscle power, agility, and endurance to make quick movements on the court [7,8]

Thus, it is crucial to recognize the essential elements of PF that are specific to each sport to develop and implement effective training programs aimed at improving individual athlete or team performance [9–11]. Identifying the suitable body composition and physiological characteristics for each sport is an essential step towards improving PF and athletic performance, particularly during adolescence when physical characteristics are maximally developed [12].

Previous studies have investigated differences in PF between different sports types in adolescent athletes, with varying results. For instance, Bencke, Damsgaard [13] found significant differences in the standing long jump between male adolescent gymnastics, swimming, handball, and tennis players, while Pion, Fransen [12] observed significant differences in the side jump among male adolescent judo, karate, and taekwondo athletes. On the other hand, Opstoel, Pion [14] found no significant differences in PF between adolescent swimmers and basketball players, and Krishnan, Sharma [15] reported no significant differences in the standing long jump among U21 fencers, weightlifters, and wrestlers. Additionally, other studies have examined the link between sports types and PF, such as football [16], volleyball [17], table tennis, softball, and baseball [18].

However, previous studies only compared statistical differences by types of sports, but did not offer the relative importance of PF elements between sports. To address this gap, we proposed a machine learning-based approach that quantifies the relative significance of physical elements in different types of sports. This approach effectively analyzes the relative importance of PF elements in five sports: track and field, football, baseball, swimming, and badminton.

## Materials and methods

### Subjects

This study included a total of 1,434 male adolescents (age: 12–18 years) who participated in five different sports, as shown in Table 1. Dataset was collected retrospectively at the Sports Science Center in Gyeonggi-do, Korea from September 2018 to October 2019. The subjects’ PF was assessed in two stages. Body composition, including body fat percentage, BMI, and weight, was measured first, followed by PF tests, which included grip strength, back muscle strength, push-up, sit-up, standing long jump, sargeant jump, side step, backward flexion, sit and reach, and eye-hand coordination (Table 2). The study was conducted according to the requirements of the Declaration of Helsinki and was approved by the Research Ethics Committee of Dankook University (IRB No. DKU 2022-02-020).

**Table 1:** Physical characteristic of the participants.

Sports types (n)	Age (yr)	Height (cm)	Weight (kg)	Fat (%)	BMI (%)
Track and field (402)	15.29±2.03	166.12±12.36	54.80±15.16	11.48±4.86	19.41±3.3
Football (537)	14.95±1.83	166.41±15.00	55.62±12.08	14.30±5.74	19.99±2.68
Baseball (328)	16.05±1.89	170.91±8.31	70.33±13.84	17.01±7.28	23.89±3.75
Swimming (105)	15.92±1.96	169.66±12.27	62.07±14.10	13.56±4.82	21.22±2.55
Badminton (62)	14.74±1.24	161.85±8.01	53.40±14.10	12.95±3.74	20.62±7.12

**Table 2:** Description of physical fitness measurement.

Physical Fitness	Elements	Description
Body composition	Body fat (%) BMI (%) Body weight (kg)	Measured with the body composition analyzer
Physical fitness	Grip strength (kg)	Recorded the right and left hand grip strength with the hand grip dynamometer
	Back muscle strength (kg)	Grab the handle of the back muscle strength meter and lift the upper body, and record the highest reading on the meter
	Push up (time/60s)	Bend the arms until the chest touches the push-up bar, and then fully extending the arms was recorded as one repetition
	Sit up (time/60s)	In the lying position, touching both elbows to both knees while keeping hands behind the neck was recorded
	Standing long jump (cm)	Jump with both feet from a starting line and record the distance between the line and the landing point of the heel
	Sargent jump (cm)	Jump vertically and measure the point where the participant touched down was recorded
	Side step (time/20s)	Measure the number of repetitions by moving the line at 1m intervals from the center line to the left and right as fast as possible
	Backward flexion (cm)	Measure the distance between the tip of the chin and the floor in a straight line when lifting the upper body as high as possible
	Sit and reach (cm)	Perform a forward bend and measure the farthest point reached by both hands
	Eye-hand coordination (time)	Record the time taken to touch 100 blue lights using the T-Wall visual reactor

### Study design

**Fig 1:**
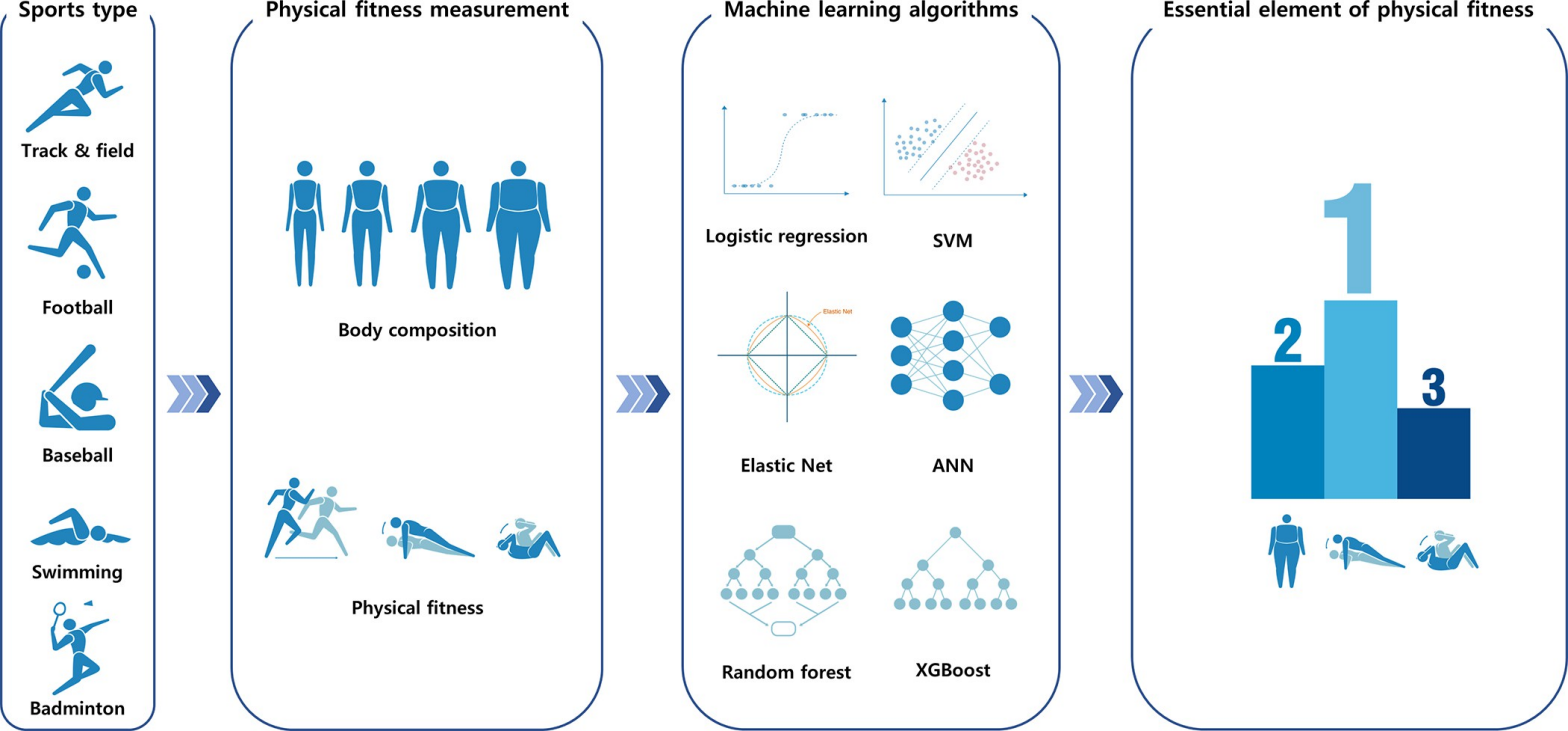
The procedure for analyzing the essential elements of physical fitness. The physical fitness of male adolescent athletes was measured by sports types, and the essential elements of physical fitness was analyzed by the developed machine learning models.

The proposed study uses PF data from male adolescent athletes across five sports types, which include track and field, football, baseball, swimming, and badminton. The study incorporates two types of data: 1) body composition data, which includes body fat percentage, BMI, and weight, and 2) PF data, which includes grip strength (GS), back muscle strength (BMS), push-up (PU), sit-up (SU), standing long jump (SLJ), sargent jump (SJ), side step (SS), backward flexion (BWF), sit and reach (SR), and eye-hand coordination (EHC). To classify two sports types, the study developed 10 machine learning models using logistic regression, support vector machine, random forest, and XGBoost. Based on these models, the study analyzed the essential elements of PF (Fig 1).

### Physical fitness measurement: Body composition

Body fat, BMI, and body weight were measured using the Inbody 720 body composition analyzer (Biospace, Korea). Participants were instructed to wear comfortable clothing and clean their palms and the soles of their feet with an electrolyte tissue. They were then asked to stand on the foot electrodes, hold the hand electrodes, and stand with both arms spread out by 30° while the measurements were taken. To minimize measurement errors, the Participants were instructed to avoid consuming meals, beverages, alcohol, and caffeine, as well as engaging in vigorous physical activity for 2 hours prior to measurement.

### Physical fitness measurement: Physical fitness

To measure PF, various components were assessed, including muscular strength (grip strength and back muscle strength), muscular endurance (push-up and sit-up), power (standing long jump and sargent jump), agility (side step), flexibility (backward flexion and sit and reach), and coordination (eye-hand coordination), as presented in Table 2.

Muscular strength was measured using a hand grip dynamometer (TKK-5401, TAKEI, Japan) for grip strength (GS) and a back muscle strength meter (TKK-5402, TAKEI, Japan) for back muscle strength (BMS). Participants stand with their feet at shoulder width and their arms hanging naturally to their sides to prevent contact with the body. Grip strength was measured twice for each hand, and the maximum value was recorded. Back muscle strength was evaluated using a back muscle strength meter (TKK-5402, TAKEI, Japan). Participants stood on a platform with their feet approximately 15 cm apart, held the handle of the strength meter with both hands, and lifted their upper body. The maximum strength was recorded from two attempts.

To assess muscular endurance, the study measured the number of push-ups and sit-ups completed by the participants. For push-ups, the participants held a push-up bar with both hands shoulder-width apart, bending their arms more than 90 degrees until their chest touched the bar, and then fully extending their arms. One repetition was counted for each full extension. For sit-ups, participants lay on their back with their hands behind their neck, touching both elbows to both knees while maintaining a 90-degree angle at the knee joint. The total number of repetitions completed in one minute was recorded for both exercises.

To assess power, the study measured the standing long jump and sargent jump. For the standing long jump, participants jumped with both feet from a starting line and the distance between the line and the landing point of the heel was measured. Two attempts were performed and the maximum distance was recorded. For the sargent jump, participants applied powder to their fingers, extended their arm upward to set the zero point, and jumped vertically. The distance in centimeters between the measuring table and the point where the participant touched down was measured. Two trials were performed, and the maximum distance was recorded for each jump.

Agility was evaluated by measuring side-step. Participants were instructed to stand in the center of three parallel lines drawn on the floor, spaced 1 meter apart. They were instructed to move as quickly as possible to the first and third lines after the starting signal sounded, then return to the center line and repeat. The total number of repetitions completed in 20 seconds was recorded.

Flexibility was measured by two tests: backward flexion and sit and reach. Backward flexion was evaluated using a backward flex meter (TKK-1860, TAKEI, Japan). Participants lay down with their hands placed behind the waist and lifted their upper body up as much as possible, while the straight-line distance from the tip of the chin to the floor was measured. The maximum value was recorded after two attempts. The Wells bench (WL-35, YAGAMI, Japan) was used for the sit and reach test. Participants sat with their legs extended and bent forward, reaching with both hands as far forward as possible and holding for 3 seconds at the point where their fingertips stopped. The maximum distance reached was recorded.

The study measured coordination through eye-hand coordination using a T-Wall visual reactor (T-Wall; 4×4, 16cell, Germany). The participants wore gloves and were timed while touching 100 blue lights with their hands. The number of errors made was also recorded. Table 2 summarizes the PF elements measured in the study and their descriptions.

### Dataset preprocessing

All features were normalized to be within the range of -1 to 1 for feature scaling. The dataset was split into a train-set, consisting of 80% of the data (322 track and field, 429 football players, 262 baseball players, 84 swimmers, and 50 badminton players), and a test-set, consisting of the remaining 20% (80 track and field, 108 football players, 60 baseball players, 21 swimmers, and 12 badminton players). The train-set was further split into 5 subsets for cross-validation [19], while ensuring that the ratio between sports types was maintained. We implemented the 5-fold cross-validation on the machine learning algorithms to select the optimal tuning parameter values, which were determined based on the highest average accuracy. Subsequently, the final model was assessed on the test-set. To address the data imbalance issue, we used the k-nearest method of SMOTE to oversample the data of the minority class so that it matches the quantity of the majority class in a 1:1 ratio [20].

### Machine learning algorithms

In this study, we used machine learning algorithms such as logistic regression (LR), support vector machine (SVM), Elastic Net (EN), artificial neural network (ANN), random forest (RF), and XGBoost (XGB) to analyze the essential elements of PF for each sports type. We developed binary classification models that selects two sports out of five sports types and classifies them, resulting in a total of 10 model results from all possible combinations. The hyperparameter optimization was performed using the random search method, and the model at the point where the accuracy was maximized after learning for 1,000 epochs was used as the final model.

LR is a method used to estimate the association between a binary dependent variable y and one or more independent variables x [21]. The hyperparameters that we optimized are regulation strength and the maximum iteration number. SVM is an algorithm that classifies data using a hyperplane that maximizes the margin between classes as a decision boundary [22]. The hyperparameters that we optimized are the regulation strength and the gaussian kernel parameter, with the optimal value being found through learning. EN is a regularization method that combines the respective advantages of L1 and L2 regulation [23]. The hyperparameters that we optimized are the alpha and L1 ratios. ANN is methods that inspired by the biological neural network in human brain [24]. The hyperparameters that we optimized are the number of hidden layers, number of units, activation function, optimizer number of epochs and batch size. RF is an ensemble method that creates a final prediction model by synthesizing results generated based on multiple decision tree models in a random manner using given data [25]. The hyperparameters that we optimized are the estimator number, maximum depth of tree, maximum number of features, minimum samples for a split, and minimum sample leaf. Finally, XGBoost is an ensemble method that combines multiple decision trees and is a boosting algorithm that can be parallelized [26]. The hyperparameters that we optimized are the estimator number, maximum depth of tree, and maximum number of features.

### Evaluation methods

A machine learning model was developed to classify two selected sports types out of five. The model was evaluated on test-set and the performance was assessed through metrics such as accuracy, precision, recall, F1-score, and the area under the receiver operating characteristic (ROC) and precision-recall (PR) curves. Furthermore, the machine learning model was utilized to analyze the essential elements of PF using feature importance of XGBoost, and SHAP (SHAPley Additional exPlanations) value methods, and the results were compared with those obtained through principal component analysis (PCA). Finally, the findings of this study were compared to previous research that examined PF differences among various sports.

PCA is a dimensionality reduction technique that involves linear transformation of high-dimensional data to low-dimensional data. [27]. The feature importance analysis method of the XGBoost, which demonstrated the highest performance, was used to evaluate the importance of each feature by determining how much it contributes to the model’s prediction, using the performance gain of the tree [28]. In addition, the SHAP value of XGBoost is a method that explains the prediction of an instance by calculating the contribution of each feature [29]. We used python (v3.7), and scikit-learn (v1.2) for development of machine learning algorithms in this study. All source codes and dataset of this study are uploaded on https://github.com/YunhwanJacobLee/Essential-elements-of-physical-fitness-analysis.

## Results

### Sports types classification performance

**Fig 2:**
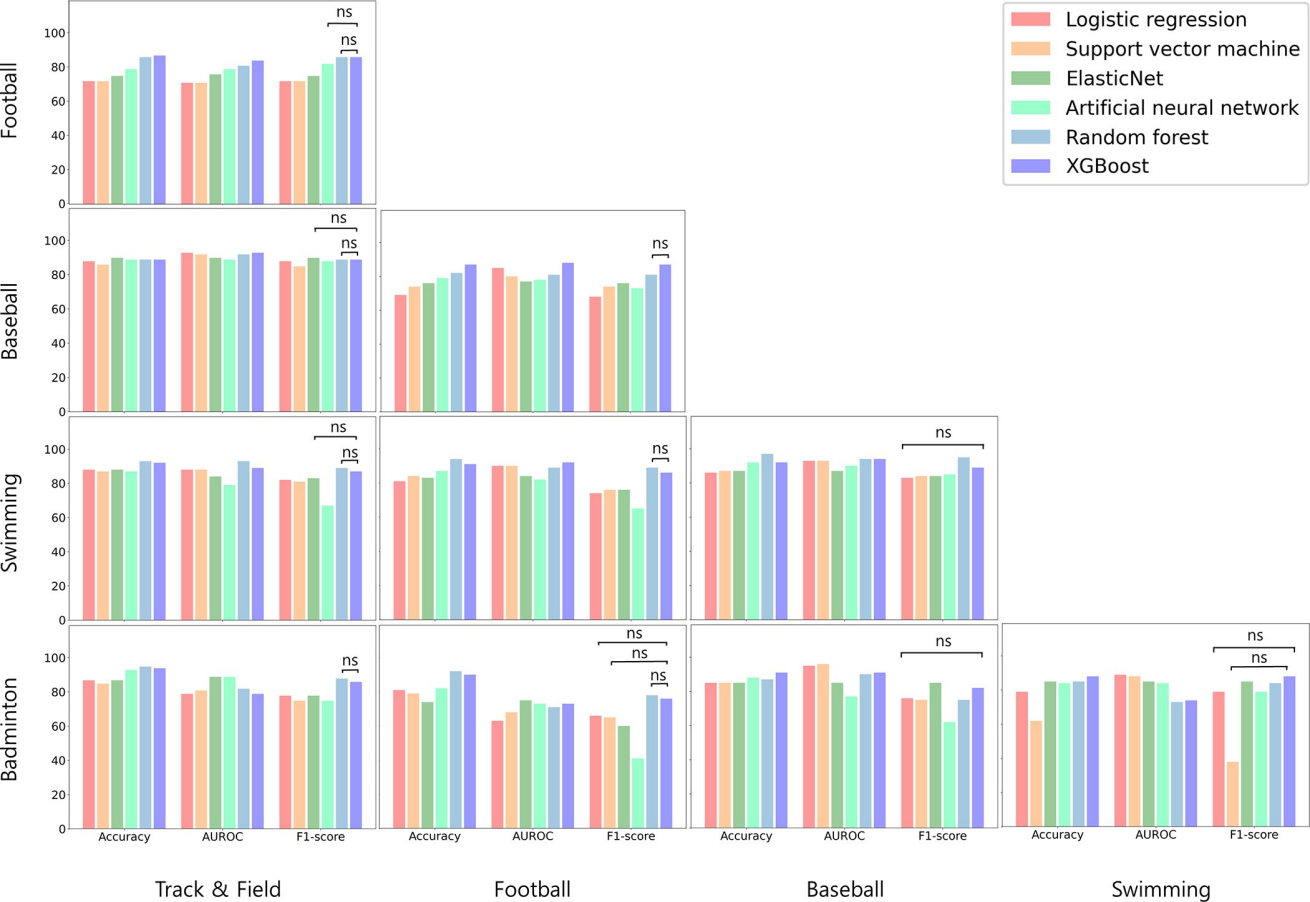
Comparison of classification performance of XGBoost by sports types: Accuracy, AUROC, F1-score.

**Fig 3:**
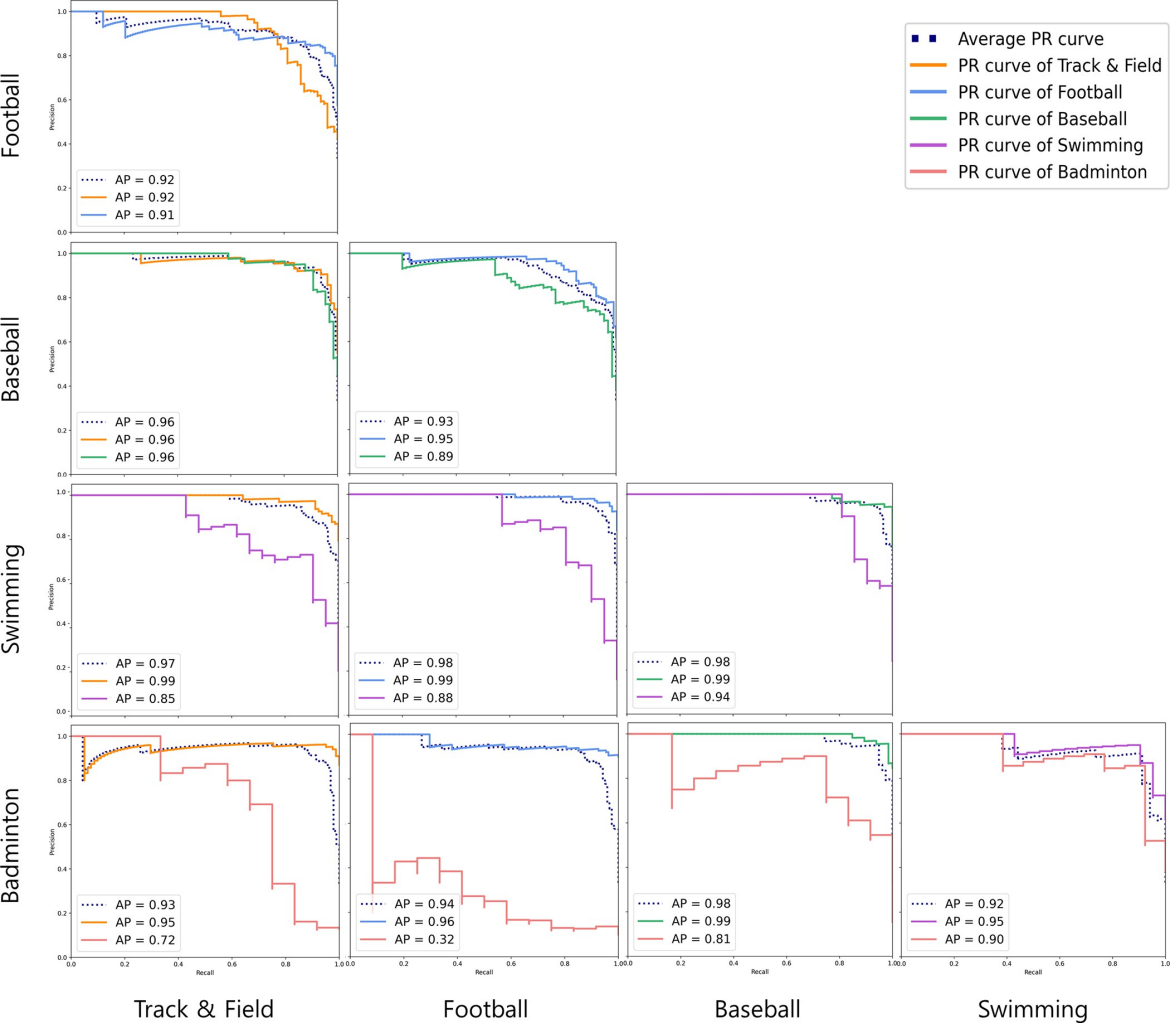
Comparison of classification performance of XGBoost by sports types: PR curve.

The study compared the performance of several machine learning algorithms and found that both random forest and XGBoost had the highest classification accuracy, with no significant difference between the two statistically (Fig 2, and S1 Table in S1 File). XGBoost was then used to classify five sports types based on Accuracy, AUROC, and F1-score. The results were as follows: 1) track & field vs. football 86.7, 0.84, 0.86; 2) track & field vs. baseball 89.04, 0.93, 0.89; 3) track & field vs. swimming 92.16, 0.89, 0.87; 4) track & field vs. badminton 93.55, 0.79, 0.86; 5) football vs. baseball 87.28, 0.88, 0.87; 6) football vs. swimming 91.47, 0.92, 0.86; 7) football vs. badminton 90, 0.73, 0.76; 8) baseball vs. swimming 91.95, 0.94, 0.98; 9) baseball vs. badminton 91.03, 0.91, 0.82; and 10) swimming vs. badminton 88.28, 0.74, 0.8, respectively (S1 Table in S1 File, Figs 2 and 3, S1 and S2 Fig).

### Analysis of the essential element of physical fitness

Through the analysis of essential elements of PF with PCA, BMS was important in track and field and football, EHC in track and field and baseball, and BF in track and field and swimming, as well as BMS in track and field and badminton, GS (avg) in football and baseball, GS (avg) in football and swimming, GS (avg) in football and badminton, BF in baseball and swimming, GS (avg) in baseball and badminton, and GS (avg) in swimming and badminton, respectively (Table 3).

**Table 3:** Comparative results of essential physical fitness analysis: 1st Principal Component Analysis (PCA), XGBoost feature importance, and SHAP value.

Sports types	1st Principal Component (PCA)	Feature Importance	SHAP Value
Track and field vs. Football	back muscle strength	standing long jump	standing long jump
Track and field vs. Baseball	eye-hand coordination	BMI	BMI
Track and field vs. Swimming	body fat	sit & reach	sit & reach
Track and field vs. Badminton	back muscle strength	standing long jump	standing long jump
Football vs. Baseball	grip strength (avg)	grip strength (L)	BMI
Football vs. Swimming	grip strength (avg)	sit up	sit up
Football vs. Badminton	grip strength (avg)	sit up	sit up
Baseball vs. Swimming	body fat	sit up	sit up
Baseball vs. Badminton	grip strength (avg)	body weight	sit up
Swimming vs. Badminton	grip strength (avg)	grip strength (L)	grip strength (L)

Through the feature importance analysis method of the XGBoost, the essential elements of PF were analyzed, and the following results were obtained: SLJ (15.9%) in track and field and football, BMI (24.1%) in track and field and baseball, SR (23.9%) in track & field and swimming, SLJ (16.6%) in track and field and badminton, GS(L) (26.2%) in football and baseball, SU (22.0%) in football and swimming, SU (20.7%) in football and badminton, SU (23.3%) in baseball and swimming, BW (56.7%) in baseball and badminton, and GS (L) (20.5%) in swimming and badminton (Table 3 and S3 Fig).

**Fig 4:**
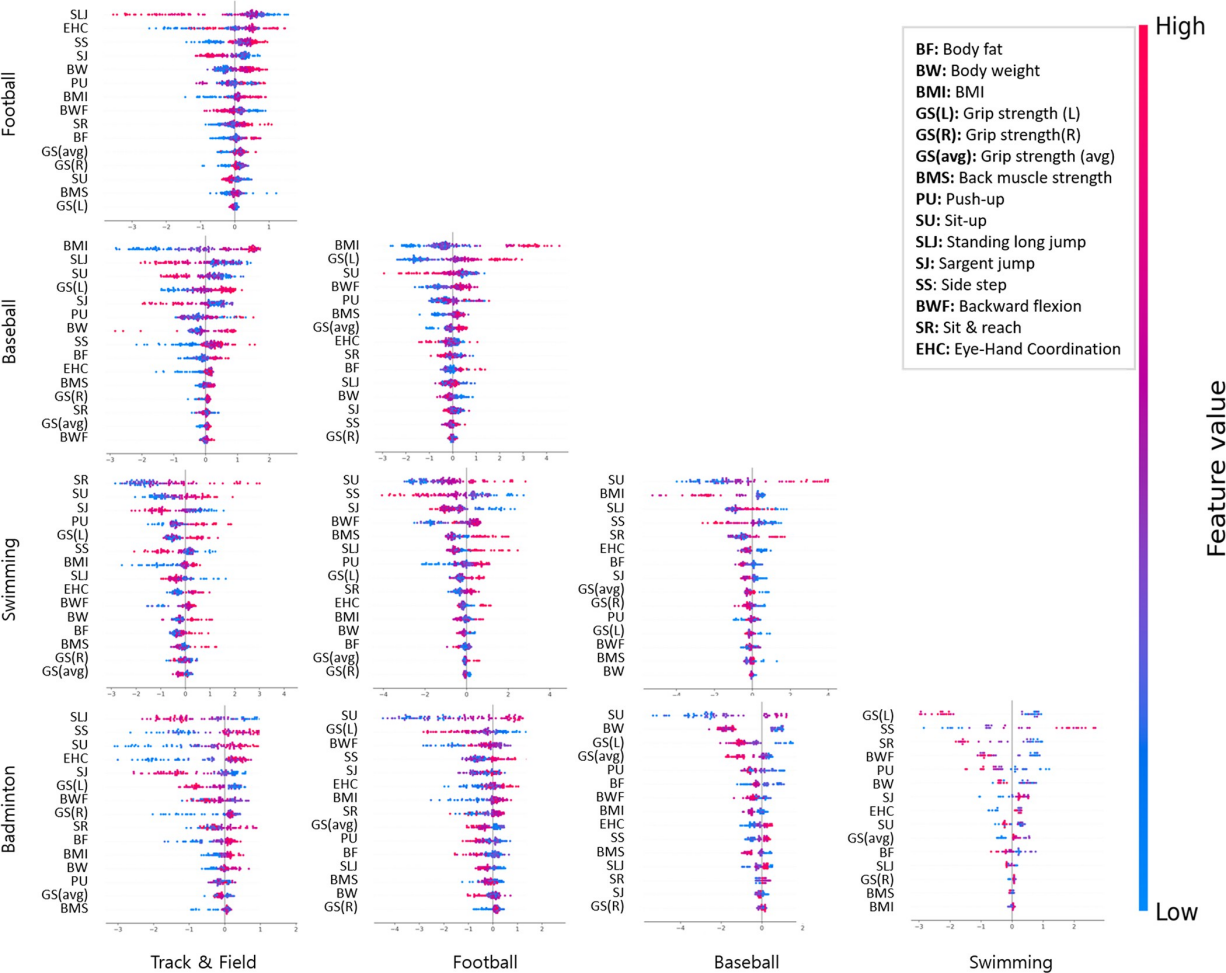
Results of analyzing the essential physical fitness of male adolescent athletes between sports types using SHAP value method. Each dot represents the influence of the physical fitness factor between sports types, where the red color of the dot indicates higher values and the blue color indicates lower values. A positive SHAP value corresponds to sports types listed vertically (football, baseball, swimming, and badminton), Negative SHAP value corresponds to sports types listed horizontally (track & field, football, baseball, and swimming) The plotted dots represent the characteristics of each individual.

The essential elements of PF were analyzed with SHAP values, showing SLJ (20.6%) in track and field and football, BMI (20.7%) in track and field and baseball, SR (21.0%) in track and field and swimming, SLJ (13.9%) in track and field and badminton, BMI (21.6%) in football and baseball, SU (19.5%) in football and swimming, SU (24.7%) in football and badminton, SU (24.8%) in baseball and swimming, SU (26.6%) in baseball and badminton, and GS (L) (19.8%) in swimming and badminton, respectively (Table 3 and Fig 4).

**Fig 5:**
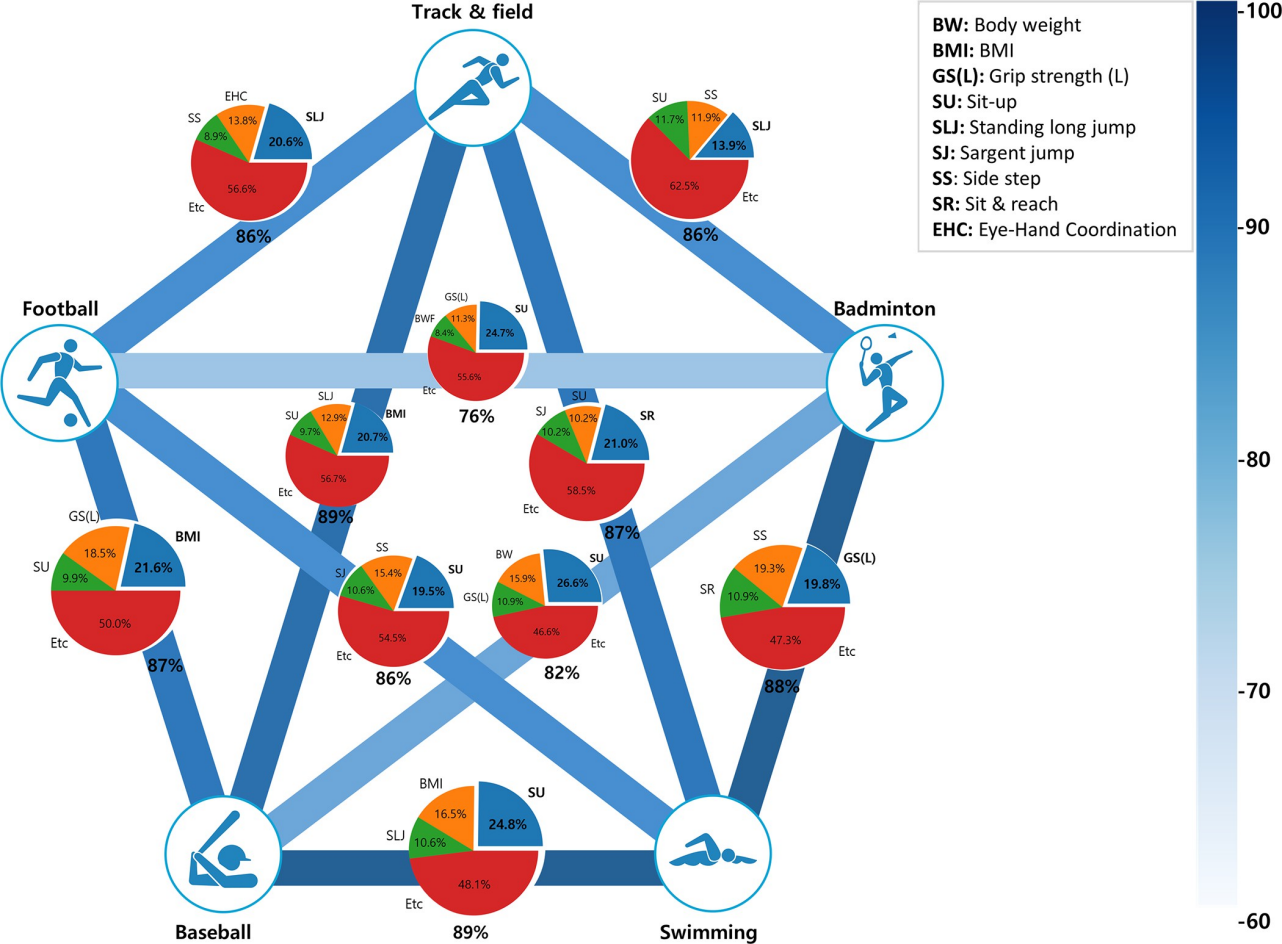
Quantitative comparison of similarity and essential elements of physical fitness between sports types using SHAP value of XGBoost. The darker the blue line between the sports types, the lower the similarity. The top 3 essential elements of physical fitness for each sports type were shown in a pie chart.

Finally, a heat map was used to show the similarity between each sports type based on the f1-score result of XGBoost (Fig 5). Additionally, a pie chart was created based on the SHAP values of XGBoost to quantitatively compare and summarize the top 3 essential elements of PF between sports types.

## Discussion

This study proposes a methodology that quantifies the relative importance of essential elements of PF and their influence between sports types by analyzing the PF profiles of male adolescent athletes using machine learning techniques. The analysis is focused on five specific sports: track & field, football, baseball, swimming, and badminton. The XGBoost model achieved an accuracy of 90.14%, an area under the receiver operating characteristic curve (AUROC) of 0.86, and an F1 score of 0.87 across the 10 analyzed sports types.

In a previous study, Elastic Net (EN) was reported to perform best in datasets with moderate samples, while XGBoost showed the highest performance in datasets with the largest number of samples [30]. In this study, however, EN showed the best performance only in track & field and baseball, while XGBoost showed higher performance in other sports types. These findings indicate results different from those reported in previous research.

Furthermore, this study employed PCA, feature importance of XGBoost and SHAP value to analyze the essential elements of PF and their impact. The PCA used is a linear analysis technique [27], and it is not suitable for analyzing the relative importance of two types of sports. Therefore, the analysis showed that grip strength is the primary factor in most sports types. Feature importance of XGBoost has a reported limitation in that it can assign high importance to features that have little effect on the model [31], whereas, SHAP value is used as a consistent feature contribution method [29]. The analysis of SHAP value method showed that certain factors had a significant impact on specific sports types, including SLJ (15.9%) for track and field and football, BMI (24.1%) for track and field and baseball, SR (23.9%) for track and field and swimming, SLJ (16.6%) for track and field and badminton, BMI (21.6%) for football and baseball, SU (22%) for football and swimming, SU (20.7%) for football and badminton, SU (23.3%) for baseball and swimming, SU (26.6%) for baseball and badminton, and SU (20.5%) for swimming and badminton. The scatter plot of the essential elements of PF and SHAP values (Fig 5) was consistent with previous studies that compared PF elements across different sports types (S1 File). In addition, the results of the feature importance of RF showed that all essential elements of PF were consistent with those of XGBoost, excluding only swimming and badminton (SU).

Moreover, this study demonstrated the utility of SHAP value method in identifying the most significant essential elements of PF and their effects on athletes in various sports types. The results of this study can assist coaches and trainers in designing more efficient exercise programs and training techniques for adolescent athletes based on their unique sports types and essential elements of PF. Additionally, the methodology used in this study can be applied to other populations and sports types to identify crucial elements of PF and their influence on athletic performance.

Developing sport-specific PF is crucial for talent development of adolescent athletes, among other factors such as genetic factors, individual characteristics, social support, and opportunities. However, previous studies have reported inconsistent results when examining the relationship between training methods and the success of youth athletes based on exercise type, and traditional statistical analyses using linear methods have been found to have limitations. As a result, recent studies have focused on using machine learning techniques to predict injury and performance, as these methods can identify complex non-linear relationships between PF data characteristics of athletes. Rommers, Rössler [32] utilized body composition and physical strength to predict injury risk in youth football players and found that peak height velocity as the most influential factor. Similarly, Musa, Majeed [33] predicted successful athletes in youth archers based on physical strength and shooting scores, and found that vertical jump and core strength were the most influential factors. However, these studies primarily aimed to predict injury and performance using machine learning techniques for specific sports types, rather than quantitatively analyzing the essential elements of PF and their influence on prediction results for each sports type. Most previous research focuses on predicting sports outcomes, such as injuries, within a single sport [34] and studies comparing the relative importance of PF across various sports predominantly use statistical methods [12,14,18]. Therefore, the analysis using the machine learning proposed in this study is, to our knowledge, the first attempt.

This study has some limitations that should be considered. Firstly, the data used in the study was collected form only one country and institution, which may limit the generalizability of the results. To improve the reliability and generalizability of the machine learning models, future studies could analyze data from multiple institutions and countries. In addition, the machine learning method we propose involves several binary classification approaches that compare sports types on a one-to-one. However, by applying multi-task learning [35], it would be possible to assess the essential elements of PF between sports types using a single model, rather than employing multiple models for one-to-one comparisons. Additionally, future research could consider a wider range of sports types, including badminton, tennis, and table tennis, to create more detailed training programs based on the essential elements of PF for various sports types. Lastly, extending the proposed method to children, adults, and women could lead to the development of specific exercise methods tailored to different populations.

## Conclusion

This study used machine learning to analyze the essential elements of physical fitness (PF) and their relative impacts on male adolescent athletes across several sports types. XGBoost demonstrated the highest performance, indicative of the similarities between two sports types. To our knowledge, this is the first attempt to quantitatively assess the relative importance of PF elements using machine learning. The proposed method can provide sports-specific recommendations for those learning sports. Furthermore, it is expected that this approach could be employed to develop personalized training methods for elite adolescent athletes tailored to their particular sports types in the future.

## Supporting information

S1 File Supporting information: Contains all the supporting tables. (DOCX)

S1 Fig Comparison of confusion matrix of XGboost by sports types. (TIF)

S2 Fig Comparison of classification performance of XGBoost by sports types: ROC curve. (TIF)

S3 Fig Results of analyzing essential physical fitness of male adolescent athletes across sports types using feature importance method of XGBoost. The red bar plot represents essential elements of physical fitness.
(TIF)
