# Supplementary material for: Essential elements of physical fitness analysis in male adolescent athletes using machine learning
Source: PLoS One. 2024 Apr 2;19(4):e0298870. doi: 10.1371/journal.pone.0298870 (PMC10986970; doi:10.1371/journal.pone.0298870)
Supplement: S1 File — (DOCX) [file pone.0298870.s001.docx]

**SUPPORTING INFORMATION**

**Comparison of machine learning analysis results and physical fitness elements between sports type**

**Comparison of track and field and football**

The proposed machine learning technique identified the standing long jump as an essential element of PF in track and field and football. The standing long jump measures power and the ability to exert maximum force in a short period of time In track and field, short ground contact time and instantaneous power to maintain stride length and flight time are critical factors [1, 2]. In contrast, football is a high-intensity intermittent exercise that runs an average of 9 to 12 km per game, and cardiorespiratory endurance such as sprinting, jogging, and walking and agility such as rapid acceleration, sudden stop, and quick change of direction are essential. [3, 4]. Vanderka, Krčmár (5) investigated the effect of jump squats on the improvement of running speed in both track and field athletes and football players improve running speed in track and field and football players, but only track and field players significantly benefited. Therefore, the standing long jump is an essential element of PF when comparing track and field and football.

**Comparison of track and field and baseball**

The proposed machine learning technique has identified BMI as an essential element of PF in track and field and baseball. BMI is an indicator of obesity in relation to weight and height [6]. In track and field, there is a correlation between BMI and running speed, with superior athletes tending to have a lower BMI [7]. In baseball, the weight of US major league players has a strong correlation with their home run record [8]. Furthermore, Crotin, Forsythe (9) reported an 11.7% increase in the rate of change of BMI among American major league hitters between 1990 and 2010. Therefore, BMI is considered an essential element of PF when comparing track and field and baseball.

**Comparison of track and field and swimming**

The proposed machine learning technique has identified sit and reach as an essential element of PF in track and field and swimming. Sit and reach is an assessment tool that measures flexibility [10]. It has been reported that lower flexibility in track and field is associated with an increase in running economy and decreases the elasticity of the muscles and tendons of the hip and leg during running [11, 12]. In swimming, higher flexibility results in smoother body movement in the water, reducing muscle resistance and energy consumption [13], Son (14) compared the flexibility of track and field athletes to that of swimmers and found that swimmers had significantly higher flexibility. Therefore, sit and reach is considered an essential element of PF when comparing track and field and swimming.

**Comparison of track and field and badminton**

The proposed machine learning technique has identified standing long jump as an essential element of PF in track and field and badminton. Track and field is characterized by straight-line running or jumping without rapid acceleration or deceleration [15, 16], and lower body strength and power during straight sprints are not related to the ability to change direction or decelerate [17]. In contrast, badminton requires repetitive motions such as hitting and dropping a shuttlecock using fast steps in a short time [18], and agility training is reported to be the most critical factor for improving athletic performance [19]. Therefore, standing long jump is considered an essential element of PF when comparing track and field and badminton.

**Comparison of football and swimming**

The proposed machine learning technique identified sit-ups as an essential element of PF in football and swimming. Sit-ups evaluate muscular endurance [20], and sports types that require muscular endurance tend to have a higher proportion of type I fibers [21]. Ricoy, Encinas (22) compared muscle fiber types in football players and swimmers, finding that football players had the highest ratio of type 2B fibers, and swimmers had the highest ratio of type 2A fibers. Kim, Chae (23) reported significantly higher sit-up performance in swimmers compared to football players. Therefore, sit-up is considered an essential element of PF when comparing football and swimming.

**Comparison of football and badminton**

The sit-up was identified as an essential element of PF in football and badminton analyzed by the proposed machine learning technique. In badminton, a complex system of 70% aerobic and 30% anaerobic is required to sustain high-intensity rallies in short periods of less than 10 seconds [24]. Compared to other racket sports like squash, and tennis, badminton was reported to have the highest intensity [25], and sit-up records were found to be the highest among other racket sports [26]. Therefore, sit-up is considered an essential element of PF when comparing football and badminton.

**Comparison of baseball and swimming**

The proposed machine learning technique analyzed sit-up as essential elements of PF in baseball and swimming. In swimming, strong body propulsion in the water requires essential contributions from the arms and hips, and core strength is essential since it is directly connected to the spine [27]. Additionally, Kim, Chae (23) (29)compared the muscular endurance of baseball, football, basketball, and swimming, and found that swimmers exhibited the highest level. Therefore, sit-up is considered an essential element of PF when comparing baseball and swimming.

**Comparison of swimming and badminton**

The proposed machine learning technique analyzed grip strength on the left side as an essential element of (PF) in swimming and badminton. In swimming, high grip strength was found to be correlated with shortened race times [28]. In racquet sports, grip strength on the dominant side is crucial for holding the racquet properly. DEFNE, Bilgehan (29), Kaplan (30) reported that badminton players exhibit significantly higher grip strength on their dominant side (50.71 kg) than on their non-dominant side (43.3 kg). Therefore, left grip strength is considered an essential element of PF when comparing swimming and badminton.

**Comparison of results of XGBoost's feature importance and SHAP value**

The sports that showed different results between XGBoost's feature importance and SHAP value were football and baseball, and baseball and badminton. First, when analyzing the essential element of PF of football and baseball using XGBoost's feature importance method, GS(L) (26.2%) was identified as the essential element of PF, while SHAP value analysis showed that BMI (21.6%) was the most important element in football and baseball. Football is closely related to agility and has no direct correlation with grip strength or performance [31]. In baseball, grip strength is closely related to an increase in pitch and bat swing speed [32, 33], and there is no significant difference between grip strength on the main and non-dominant sides due to the frequent use of the non-dominant hand when wearing gloves and holding a baseball bat [34]. Previous studies have reported a negative effect of BMI on football performance [35], with the average BMI of European professional football players in Spain, England, Germany, and Italy being around 23 [36]. On the other hand, high body weight has been found to be positively associated with better baseball performance, and the rate of change in BMI continuously increases [9].

The essential element of PF in football and baseball was found to be weight (56.7%) according to the results obtained from XGBoost's feature importance method, while sit-up (26.6%) was identified as essential using SHAP value analysis. In baseball, performance is related to the amount of body fat, with higher levels of fat increasing power for pitching and hitting, and a larger body size being optimal for performance [8, 37]. In badminton, quick movements such as jumps, lunges, and changes of direction are required, and excess body weight can limit these movements and strokes [38, 39]. Additionally, badminton demands a complex muscular endurance system with 70% aerobic and 30% anaerobic properties to sustain high-intensity rallies that last less than 10 seconds [24]. In contrast, physical strength in baseball players is related to pitch and batted ball speed, BMI, and grip strength, but not to sit-up [32].

| **S1 Table. The performance comparison of machine learning algorithms for classifying five different sports types in male adolescent athletes.** | | | | | | | | |
| --- | --- | --- | --- | --- | --- | --- | --- | --- |
| **No.** | **Sports types** | **Machine learning algorithm** | **Accuracy**  **(%)** | **AUROC** | **F1-score** | **Spec** | **Sens** | **p-value of**  **F1-score**  **(vs. XGB)** |
| 1 | Track & field vs.  Football | LR | 72.34 | 0.71 | 0.72 | 0.78 | 0.70 | 0.001* |
|  |  | SVM | 71.81 | 0.71 | 0.72 | 0.80 | 0.66 | 0.001* |
|  |  | EN | 75.00 | 0.76 | 0.75 | 0.80 | 0.71 | 0.003* |
|  |  | ANN | 79.26 | 0.79 | 0.82 | 0.74 | 0.81 | 0.283 |
|  |  | RF | 86.17 | 0.81 | **0.86** | 0.86 | **0.86** | 0.408 |
|  |  | XGB | **86.70** | **0.84** | **0.86** | **0.89** | 0.84 |  |
| 2 | Track & field vs.  Baseball | LR | 88.37 | 0.93 | 0.88 | 0.89 | 0.83 | 0.433 |
|  |  | SVM | 85.62 | 0.92 | 0.85 | 0.89 | 0.85 | 0.504 |
|  |  | EN | **90.41** | 0.90 | **0.90** | 0.93 | **0.86** | 0.823 |
|  |  | ANN | 89.32 | 0.89 | 0.88 | **0.95** | 0.83 | 0.521 |
|  |  | RF | **89.04** | 0.92 | 0.89 | 0.93 | 0.85 | 0.718 |
|  |  | XGB | **89.04** | **0.93** | **0.89** | 0.9 | 0.83 |  |
| 3 | Track & field vs. Swimming | LR | 88.24 | 0.88 | 0.82 | 0.93 | 0.71 | 0.052 |
|  |  | SVM | 87.25 | 0.88 | 0.81 | 0.91 | 0.67 | 0.015* |
|  |  | EN | 88.24 | 0.84 | 0.83 | 0.91 | **0.76** | 0.220 |
|  |  | ANN | 86.67 | 0.79 | 0.67 | 0.92 | 0.65 | 0.001* |
|  |  | RF | **93.14** | **0.93** | **0.89** | **1.0** | 0.71 | 0.772 |
|  |  | XGB | 92.16 | 0.89 | 0.87 | 0.98 | 0.67 |  |
| 4 | Track & field vs. Badminton | LR | 87.09 | 0.79 | 0.78 | 0.86 | 0.92 | 0.032* |
|  |  | SVM | 84.95 | 0.81 | 0.75 | 0.85 | 0.83 | 0.007* |
|  |  | EN | 87.09 | **0.89** | 0.78 | 0.86 | 0.91 | 0.032* |
|  |  | ANN | 92.69 | 0.89 | 0.75 | 0.94 | **0.83** | 0.007* |
|  |  | RF | **94.62** | 0.82 | **0.88** | 0.95 | 0.83 | 0.174 |
|  |  | XGB | 93.55 | 0.79 | 0.86 | **0.96** | 0.83 |  |
| 5 | Football  vs.  Baseball | LR | 68.79 | 0.85 | 0.68 | 0.64 | 0.76 | 0.020* |
|  |  | SVM | 73.99 | 0.8 | 0.74 | 0.69 | 0.80 | 0.002* |
|  |  | EN | 76.30 | 0.77 | 0.76 | 0.73 | 0.82 | 0.010* |
|  |  | ANN | 79.77 | 0.78 | 0.73 | 0.84 | 0.72 | 0.002* |
|  |  | RF | 82.08 | 0.81 | 0.81 | 0.83 | 0.78 | 0.144 |
|  |  | XGB | **87.28** | **0.88** | **0.87** | **0.87** | **0.86** |  |
| 6 | Football  vs. Swimming | LR | 81.39 | 0.9 | 0.74 | 0.80 | 0.86 | 0.001* |
|  |  | SVM | 83.72 | 0.9 | 0.76 | 0.83 | 0.86 | 0.002* |
|  |  | EN | 82.95 | 0.84 | 0.76 | 0.82 | 0.86 | 0.001* |
|  |  | ANN | 86.67 | 0.82 | 0.65 | 0.89 | 0.76 | 0.000* |
|  |  | RF | **93.79** | 0.89 | **0.89** | 0.95 | **0.90** | 0.818 |
|  |  | XGB | 91.47 | **0.92** | 0.86 | 0.94 | **0.90** |  |
| 7 | Football  vs. Badminton | LR | 80.83 | 0.63 | 0.66 | 0.81 | 0.75 | 0.274 |
|  |  | SVM | 79.17 | 0.68 | 0.65 | 0.79 | **0.83** | 0.253 |
|  |  | EN | 74.17 | 0.75 | 0.60 | 0.74 | 0.75 | 0.002 |
|  |  | ANN | 82.33 | 0.73 | 0.41 | 0.84 | 0.62 | 0.000 |
|  |  | RF | **91.67** | 0.71 | **0.78** | **0.97** | 0.42 | 0.594 |
|  |  | XGB | 90.00 | **0.73** | 0.76 | 0.94 | 0.67 |  |
| 8 | Baseball  vs. Swimming | LR | 86.21 | 0.93 | 0.83 | 0.86 | 0.86 | 0.124 |
|  |  | SVM | 87.36 | 0.93 | 0.84 | 0.92 | 0.86 | 0.196 |
|  |  | EN | 87.36 | 0.87 | 0.84 | 0.88 | 0.86 | 0.174 |
|  |  | ANN | 92.18 | 0.90 | 0.85 | 0.95 | 0.86 | 0.222 |
|  |  | RF | **96.55** | 0.94 | **0.95** | **0.97** | **0.95** | 0.946 |
|  |  | XGB | 91.95 | **0.94** | 0.89 | 0.95 | 0.81 |  |
| 9 | Baseball  vs. Badminton | LR | 84.62 | 0.95 | 0.76 | 0.84 | 0.83 | 0.079 |
|  |  | SVM | 84.62 | 0.96 | 0.75 | **0.89** | 0.67 | 0.059 |
|  |  | EN | 85.29 | 0.85 | 0.85 | 0.86 | **0.85** | 0.128 |
|  |  | ANN | 88.46 | 0.77 | 0.62 | **0.94** | 0.60 | 0.001* |
|  |  | RF | 87.18 | 0.90 | 0.75 | 0.91 | 0.58 | 0.103 |
|  |  | XGB | **91.03** | **0.91** | **0.82** | **0.95** | 0.75 |  |
| 10 | Swimming vs. Badminton | LR | 79.41 | 0.89 | 0.79 | 0.76 | 0.85 | 0.169 |
|  |  | SVM | 61.76 | 0.88 | 0.38 | **1** | 0 | 0.000* |
|  |  | EN | 85.29 | 0.85 | 0.85 | 0.86 | 0.85 | 0.279 |
|  |  | ANN | 84.12 | 0.84 | 0.79 | 0.85 | 0.83 | 0.156 |
|  |  | RF | 85.29 | 0.73 | 0.84 | **0.90** | 0.77 | 0.345 |
|  |  | XGB | **88.24** | 0.74 | **0.88** | **0.90** | **0.85** |  |

| **S2 Table. The performance of Random Forest and XGBoost changed after applying SMOTE, compared to their baseline performance prior to the application.** | | | | | | | |
| --- | --- | --- | --- | --- | --- | --- | --- |
| **No.** | **Sports types** | **Machine learning algorithm** | **Accuracy**  **(%)** | **AUROC** | **F1-score** | **Specificity** | **Sensitivity** |
| 1 | Track & field  vs.  Football | RF | 86.17 (-4.79) | 0.81 (0.00) | 0.86 (-0.05) | 0.86 (-0.1) | 0.86 (-0.01) |
|  |  | XGB | 86.70 (-1.59) | 0.84 (+0.01) | 0.86 (-0.01) | 0.89 (-0.04) | 0.84 (+0.01) |
| 2 | Track & field  vs.  Baseball | RF | 89.04 (+0.69) | 0.92 (-0.03) | 0.89 (0.00) | 0.93 (+0.03) | 0.85 (-0.03) |
|  |  | XGB | 86.17 (+2.87) | 0.86 (+0.07) | 0.86 (+0.03) | 0.86 (+0.04) | 0.86 (-0.03) |
| 3 | Track & field  vs.  Swimming | RF | 93.14 (-1.96) | 0.93 (-0.14) | 0.89 (-0.05) | 1.0 (0.00) | 0.71 (-0.14) |
|  |  | XGB | 92.16 (-0.98) | 0.89 (-0.09) | 0.87 (-0.03) | 0.98 (0.00) | 0.67 (-0.14) |
| 4 | Track & field  vs.  Badminton | RF | 94.62 (-2.15) | 0.82 (-0.11) | 0.88 (-0.11) | 0.95 (+0.05) | 0.83 (-0.41) |
|  |  | XGB | 93.55 (0.00) | 0.79 (-0.04) | 0.86 (-0.04) | 0.96 (+0.04) | 0.83 (-0.33) |
| 5 | Football  vs.  Baseball | RF | 82.08 (+1.74) | 0.81 (0.00) | 0.81 (+0.01) | 0.83 (+0.09) | 0.78 (-0.06) |
|  |  | XGB | 87.28 (-0.58) | 0.88 (-0.02) | 0.87 (0.00) | 0.87 (+0.06) | 0.86 (-0.06) |
| 6 | Football  vs.  Swimming | RF | 93.79 (-3.1) | 0.89 (-0.1) | 0.89 (-0.08) | 0.95 (+0.01) | 0.90 (-0.28) |
|  |  | XGB | 91.47 (+2.32) | 0.92 (-0.05) | 0.86 (+0.02) | 0.94 (-0.03) | 0.90 (-0.14) |
| 7 | Football  vs.  Badminton | RF | 91.67 (-0.84) | 0.71 (-0.17) | 0.78 (-0.23) | 0.97 (+0.03) | 0.42 (-0.34) |
|  |  | XGB | 90.00 (+3.33) | 0.73 (+0.03) | 0.76 (0.00) | 0.94 (+0.05) | 0.67 (-0.25) |
| 8 | Baseball  vs.  Swimming | RF | 96.55 (-2.3) | 0.94 (-0.03) | 0.95 (-0.03) | 0.97 (0.00) | 0.95 (-0.09) |
|  |  | XGB | 91.95 (+3.45) | 0.94 (+0.02) | 0.89 (+0.05) | 0.95 (+0.03) | 0.81 (+0.05) |
| 9 | Baseball  vs.  Badminton | RF | 87.18 (+3.85) | 0.90 (-0.12) | 0.75 (+0.06) | 0.91 (+0.06) | 0.58 (0.00) |
|  |  | XGB | 91.03 (+3.84) | 0.91 (-0.01) | 0.82 (+0.08) | 0.95 (+0.02) | 0.75 (+0.08) |
| 10 | Swimming  vs.  Badminton | RF | 85.29 (0.00) | 0.73 (+0.09) | 0.84 (0.00) | 0.90 (+0.05) | 0.77 (-0.08) |
|  |  | XGB | 88.24 (0.00) | 0.74 (+0.14) | 0.88 (0.00) | 0.90 (0.00) | 0.85 (0.00) |
| RF : Random forest, XGB : XGBoost | | | | | | | |

| **S3 Table. The skewness and kurtosis changed after the application of SMOTE compared to the baseline values before its application.** | | | | | | | | | | | | | | | | | |
| --- | --- | --- | --- | --- | --- | --- | --- | --- | --- | --- | --- | --- | --- | --- | --- | --- | --- |
| **No.** | **Sports types** |  | **BF** | **BW** | **BMI** | **GS (L)** | **GS (R)** | **GS (avg)** | **BMS** | **PU** | **SU** | **SLJ** | **SJ** | **SS** | **BWF** | **SR** | **EHC** |
| 1 | Track & field  vs.  Football | Skew | 1.55  (-0.04) | 0.62  (+0.03) | 1.27  (+0.00) | 0.17  (+0.04) | 0.23  (+0.07) | 0.16  (+0.15) | 0.31  (+0.06) | 0.36  (+0.03) | -0.28  (-0.01) | -0.41  (-0.78) | -0.16  (+0.01) | -1.17  (-0.17) | 14.79  (-0.11) | -0.29  (-0.01) | 0.84  (-0.12) |
|  |  | Kurt | 3.78  (-0.34) | 7.74  (+5.98) | 4.02  (-0.11) | -0.46  (+0.04) | -0.39  (+0.1) | -0.44  (+0.06) | -0.41  (+0.11) | 0.15  (+0.01) | 0.32  (-0.03) | 2.51  (+0.68) | 1.54  (+0.33) | 8.38  (+0.53) | 330.77  (-18.79) | 0.02  (+0.11) | 1.84  (-0.38) |
| 2 | Track & field  vs.  Baseball | Skew | 1.30  (+0.06) | 0.25  (+0.06) | 0.76  (+0.08) | 0.04  (+0.03) | -0.15  (+0.01) | -0.10  (+0.02) | 0.04  (0.00) | 0.49  (+0.01) | -0.16  (-0.02) | -0.19  (-0.01) | -0.08  (-0.01) | -0.58  (0.00) | -0.36  (-0.01) | 0.01  (-0.01) | 1.12  (-0.01) |
|  |  | Kurt | 1.55  (+0.24) | 0.08  (-0.01) | 0.23  (+0.09) | -0.12  (-0.01) | -0.43  (-0.05) | -0.27  (-0.04) | -0.57  (-0.03) | 0.22  (+0.1) | 0.19  (-0.09) | -0.39  (-0.07) | -0.29  (-0.05) | 1.21  (-0.06) | 0.21  (+0.05) | 1.72  (-0.01) | 2.67  (-0.2) |
| 3 | Track & field  vs.  Swimming | Skew | 1.89  (+0.15) | 0.64  (+0.35) | 1.36  (+0.42) | -0.01  (+0.21) | 0.06  (+0.1) | 0.04  (+0.12) | 0.24  (+0.13) | 0.35  (+0.00) | -0.36  (+0.19) | -0.40  (+0.1) | -0.31  (+0.12) | -0.35  (-0.12) | -0.34  (+0.09) | -0.17  (+0.14) | 0.96  (+0.11) |
|  |  | Kurt | 5.55  (+0.41) | 1.29  (+0.81) | 3.85  (+1.0) | -0.69  (+0.14) | -0.77  (0.17) | -0.77  (+0.22) | -0.82  (+0.26) | -0.01  (+0.3) | 0.58  (-0.14) | -0.56  (+0.09) | -0.29  (-0.2) | 0.45  (+0.21) | 0.19  (-0.13) | 0.11  (+0.12) | 1.52  (+0.42) |
| 4 | Track & field  vs.  Badminton | Skew | 1.89  (+0.4) | 1.25  (-0.18) | 5.79  (+0.2) | 0.20  (-0.32) | 0.15  (-0.24) | 0.15  (-0.29) | 0.37  (-0.24) | 0.17  (-0.05) | -0.30  (+0.19) | -0.17  (-0.41) | -0.12  (-0.4) | -0.44  (+0.09) | -0.19  (-0.32) | -0.51  (+0.11) | 0.94  (+0.2) |
|  |  | Kurt | 5.65  (+0.43) | 4.02  (-1.11) | 63.31  (+10.82) | -0.45  (-0.46) | -0.55  (-0.49) | -0.48  (-0.46) | -0.49  (-0.5) | -0.21  (+0.11) | 0.36  (-0.52) | -0.65  (-0.06) | -0.5  (+0.01) | 0.77  (-0.47) | 0.26  (-0.67) | 0.19  (-0.65) | 1.76  (-0.11) |
| 5 | Football  vs.  Baseball | Skew | 1.82  (+0.73) | 0.11  (+0.06) | 0.65  (+0.12) | 0.16  (+0.14) | 0.13  (+0.12) | 0.11  (+0.13) | 0.22  (+0.08) | 0.64  (+0.05) | -0.24  (+0.09) | -0.29  (+0.03) | 0.10  (+0.08) | -0.41  (+0.08) | -0.16  (+0.09) | 0.20  (+0.1) | 0.73  (-0.06) |
|  |  | Kurt | 1.76  (+0.45) | -0.19  (-0.01) | 0.22  (+0.28) | -0.42  (-0.01) | -0.52  (-0.01) | -0.5  (-0.02) | -0.33  (+0.02) | 0.24  (+0.2) | 0.39  (-0.23) | -0.01  (+0.17) | 0.03  (+0.02) | 0.73  (-0.39) | -0.03  (-0.06) | 1.82  (+0.05) | 1.21  (-0.49) |
| 6 | Football  vs.  Swimming | Skew | 1.39  (-0.18) | 0.01  (+0.09) | 0.25  (+0.17) | 0.21  (+0.23) | 0.36  (+0.15) | 0.29  (+0.18) | 0.39  (+0.2) | 0.55  (+0.26) | -0.19  (+0.26) | -0.18  (+0.05) | 0.09  (+0.15) | -0.20  (-0.16) | -0.09  (+0.3) | 0.10  (+0.12) | 0.58  (+0.01) |
|  |  | Kurt | 3.42  (-1.43) | -0.58  (+0.33) | -0.23  (+0.51) | -0.72  (+0.28) | -0.48  (+0.46) | -0.62  (+0.98) | -0.51  (+0.59) | 0.05  (+0.53) | 0.29  (+0.28) | -0.17  (+0.39) | -0.21  (+0.3) | -0.29  (+0.24) | -0.03  (-0.1) | 0.12  (+0.45) | 0.33  (+0.25) |
| 7 | Football  vs.  Badminton | Skew | 1.32  (+0.03) | 0.59  (-0.18) | 7.05  (+0.83) | 0.26  (-0.13) | 0.27  (-0.08) | 0.24  (-0.12) | 0.30  (-0.09) | 0.57  (+0.06) | -0.22  (+0.2) | -0.34  (-0.16) | 0.14  (-0.08) | -0.19  (+0.1) | 0.02  (-0.09) | -0.18  (+0.09) | 0.66  (+0.09) |
|  |  | Kurt | 3.26  (-0.63) | 3.33  (-0.54) | 102.69  (+30.27) | -0.54  (-0.28) | -0.45  (-0.13) | -0.53  (-0.17) | -0.45  (-0.06) | 0.4  (-0.07) | 0.34  (-0.42) | 0.02  (+0.34) | -0.03  (+0.06) | -0.1  (-0.07) | 0.24  (-0.7) | 0.07  (-0.37) | 0.62  (+0.2) |
| 8 | Baseball  vs.  Swimming | Skew | 0.95  (-0.31) | -0.21  (+0.06) | 0.42  (-0.15) | -0.23  (+0.13) | -0.29  (-0.02) | -0.37  (+0.06) | -0.19  (-0.01) | 0.33  (+0.16) | -0.09  (+0.14) | -0.43  (+0.06) | -0.24  (+0.09) | -0.53  (-0.17) | -0.27  (-0.05) | 0.41  (+0.32) | 0.77  (-0.11) |
|  |  | Kurt | 0.49  (-1.1) | 0.12  (+0.2) | -0.28  (-0.47) | 0.25  (+0.23) | -0.18  (+0.4) | -0.01  (+0.35) | -0.3  (+0.52) | -0.49  (-0.12) | 0.56  (+0.31) | -0.34  (+0.21) | 0.09  (+0.13) | 0.5  (+0.27) | 0.19  (-0.06) | 1.43  (+1.09) | 1.25  (+0.26) |
| 9 | Baseball  vs.  Badminton | Skew | 0.89  (-0.44) | 0.03  (-0.55) | 0.38  (-0.48) | -0.01  (-0.48) | -0.13  (-0.29) | -0.11  (-0.4) | 0.02  (-0.34) | 0.53  (-0.12) | -0.29  (+0.37) | -0.25  (-0.11) | -0.04  (-0.18) | -0.59  (+0.19) | -0.09  (-0.4) | 0.58  (+0.05) | 0.71  (+0.19) |
|  |  | Kurt | 0.34  (-1.64) | -0.16  (-0.22) | -0.47  (-0.79) | -0.07  (+0.01) | -0.05  (+0.08) | -0.05  (+0.11) | -0.2  (+0.03) | -0.16  (-0.61) | 0.66  (-0.21) | -0.48  (+0.2) | 0.23  (+0.1) | 0.93  (-0.69) | -0.06  (-0.08) | 3.88  (-1.02) | 1.7  (+0.53) |
| 10 | Swimming  vs.  Badminton | Skew | 1.20  (-0.01) | 1.07  (-0.54) | 7.53  (+2.08) | 0.09  (-0.16) | 0.23  (-0.16) | 0.18  (-0.17) | 0.29  (-0.14) | 0.25  (-0.14) | -0.74  (-0.11) | -0.16  (-0.18) | -0.11  (-0.15) | -0.19  (+0.15) | 0.19  (-0.15) | 0.04  (-0.19) | 0.37  (+0.08) |
|  |  | Kurt | 3.01  (-0.4) | 4.75  (-0.81) | 74.92  (+41.45) | -1.09  (-0.15) | -0.91  (-0.22) | -1.08  (-0.24) | -1.03  (-0.22) | -0.67  (-0.22) | 2.94  (-0.19) | -1.05  (0.00) | -0.77  (+0.01) | -0.81  (-0.25) | -1.05  (-0.11) | -0.18  (-0.09) | -0.81  (-0.08) |
| Skew : skewness, Kurt : kurtosis, BF : body fat, BW : body weight, BMI : BMI, GS(L) : grip strength(L), GS(R) : grip strength(R), GS(avg) : grip strength(avg), BMS : back muscle strength, PU : push-up, SU : sit-up, SLJ : standing long jump, SJ : sargent jump, SS : side step, BWF : backward flexion, SR : sit & reach, EHC : eye-hand coordination | | | | | | | | | | | | | | | | | |

**REFERENCES**

1. Morin J, Belli A. Mechanical factors of 100 m sprint performance in trained athletes. SCIENCE ET SPORTS. 2003;18(3):161-3.

2. Faccioni A. Relationships between selected speed strength performance tests and temporal variables of maximal running velocity: University of Canberra; 1995.

3. Impellizzeri F, Rampinini E, Castagna C, Bishop D, Bravo DF, Tibaudi A, et al. Validity of a repeated-sprint test for football. International journal of sports medicine. 2008;29(11):899-905.

4. Reilly T, Williams AM, Nevill A, Franks A. A multidisciplinary approach to talent identification in soccer. Journal of sports sciences. 2000;18(9):695-702.

5. Vanderka M, Krčmár M, Longová K, Walker S. Acute effects of loaded half-squat jumps on sprint running speed in track and field athletes and soccer players. Journal of strength and conditioning research. 2016;30(6):1540-6.

6. Stevens J, McClain J, Truesdale K. Selection of measures in epidemiologic studies of the consequences of obesity. International Journal of Obesity. 2008;32(3):S60-S6.

7. Marc A, Sedeaud A, Guillaume M, Rizk M, Schipman J, Antero-Jacquemin J, et al. Marathon progress: demography, morphology and environment. Journal of sports sciences. 2014;32(6):524-32.

8. Hamburg L, Hines TM. Correlations for weight, height and two measures of batting performance. Perceptual and motor skills. 1999;88(2):466-8.

9. Crotin RL, Forsythe CM, Bhan S, Karakolis T. Changes in physical size among major league baseball players and its attribution to elite offensive performance. The Journal of Strength & Conditioning Research. 2014;28(10):2705-8.

10. Hui S-C, Yuen PY. Validity of the modified back-saver sit-and-reach test: a comparison with other protocols. Medicine and science in sports and exercise. 2000;32(9):1655-9.

11. Jones AM. Running economy is negatively related to sit-and-reach test performance in international-standard distance runners. International journal of sports medicine. 2002;23(01):40-3.

12. Craib MW, Mitchell VA, Fields KB, Cooper TR, Hopewell R, Morgan D. The association between flexibility and running economy in sub-elite male distance runners. Medicine and science in sports and exercise. 1996;28(6):737-43.

13. Maglischo EW. Swimming fastest: Human kinetics; 2003.

14. Son H-J. Comparison of Physical Characteristics and Physical Fitness between High School Elite Triathlon Athletes and Mid- and Long-distance Athletics and Swimmers. The Korea Journal of Sports. 2020;18(1):845-52.

15. Jones PA, Bampouras T, Marrin K. An investigation into the physical determinants of change of direction speed. Journal of Sports Medicine and Physical Fitness. 2009;49(1):97-104.

16. Salaj S, Markovic G. Specificity of jumping, sprinting, and quick change-of-direction motor abilities. The Journal of Strength & Conditioning Research. 2011;25(5):1249-55.

17. Young WB, McDowell MH, Scarlett BJ. Specificity of sprint and agility training methods. The Journal of Strength & Conditioning Research. 2001;15(3):315-9.

18. Manrique DC, Gonzalez-Badillo J. Analysis of the characteristics of competitive badminton. British journal of sports medicine. 2003;37(1):62-6.

19. Tiwari L, Rai V, Srinet S. Relationship of selected motor fitness components with the performance of badminton player. Asian J Phys Educ Comput Sci Sports. 2011;5(1):88-91.

20. Kloubec JA. Pilates for improvement of muscle endurance, flexibility, balance, and posture. The Journal of Strength & Conditioning Research. 2010;24(3):661-7.

21. Lindle R, Metter E, Lynch N, Fleg Jv, Fozard J, Tobin J, et al. Age and gender comparisons of muscle strength in 654 women and men aged 20–93 yr. Journal of applied physiology. 1997;83(5):1581-7.

22. Ricoy J, Encinas A, Cabello A, Madero S, Arenas J. Histochemical study of the vastus lateralis muscle fibre types of athletes. Journal of physiology and biochemistry. 1998;54(1):41-7.

23. Kim K, Chae J, Cho H, Kim H. A Stusy on a Spcifics of Physical Fitness in Middle School Athletes. Journal of the Korea Exercise Science 1998;7(2):207-20.

24. Chin M-K, Wong AS, So RC, Siu OT, Steininger K, Lo D. Sport specific fitness testing of elite badminton players. British journal of sports medicine. 1995;29(3):153-7.

25. Cabello D, Tobar H, Puga E, Delgado M. Determinación del metabolismo energético en bádminton. Archivos de Medicina del Deporte. 1997;62:469-75.

26. Choi IB. The Study on a Body Composition and Physical Fitness Characteristics of Racket Players. The Korean Journal of Sports Science. 2003;12(1):367-78.

27. Fig G. Strength training for swimmers: Training the core. Strength & Conditioning Journal. 2005;27(2):40-2.

28. Zampagni ML, Casino D, Benelli P, Visani A, Marcacci M, De Vito G. Anthropometric and strength variables to predict freestyle performance times in elite master swimmers. The Journal of Strength & Conditioning Research. 2008;22(4):1298-307.

29. DEFNE Ö, Bilgehan B, TUBA M. Comparison of anthropometric measurements of dominant hands between adult elite volleyball players and sedentaries. Ovidius University Annals, Series Physical Education and Sport/Science, Movement and Health. 2010;10(2):546-9.

30. Kaplan DÖ. Evaluating the Relation between Dominant and Non-Dominant Hand Perimeters and Handgrip Strength of Basketball, Volleyball, Badminton and Handball Athletes. International Journal of Environmental and Science Education. 2016;11(10):3297-309.

31. James RS, Thake CD, Birch SL. Relationships between measures of physical fitness change when age-dependent bias is removed in a group of young male soccer players. The Journal of Strength & Conditioning Research. 2017;31(8):2100-9.

32. Nakata H, Nagami T, Higuchi T, Sakamoto K, Kanosue K. Relationship between performance variables and baseball ability in youth baseball players. The Journal of Strength & Conditioning Research. 2013;27(10):2887-97.

33. Crotin R, Ramsey D. Grip Strength Measurement in Baseball Pitchers: A Clinical Examination to Indicate Stride Length Inefficiency. International Journal of Sports Physical Therapy. 2021;16(5):1330.

34. Jarit P. Dominant-hand to nondominant-hand grip-strength ratios of college baseball players. Journal of Hand Therapy. 1991;4(3):123-6.

35. Taghinejad S. Relationship between anthropometric measures (weight, height, body mass index) with some elements of physical fitness (agility, speed, balance) in girls ages 12 to 14: Master thesis, Islamic Azad University; 2013.

36. Bloomfield J, Polman R, Butterly R, O’Donoghue P. Analysis of age, stature, body mass, BMI and quality of elite soccer players from 4 European Leagues. J Sports Med Phys Fitness. 2005;45(1):58-67.

37. Szymanski DJ. Physiology of baseball pitching dictates specific exercise intensity for conditioning. Strength & Conditioning Journal. 2009;31(2):41-7.

38. Kuntze G, Mansfield N, Sellers W. A biomechanical analysis of common lunge tasks in badminton. Journal of sports sciences. 2010;28(2):183-91.

39. Reilly T, Secher N, Snell P, Williams C, Williams C. Physiology of sports: Routledge; 2005.
