# Supplementary figures and images for: Essential elements of physical fitness analysis in male adolescent athletes using machine learning
Source: PLoS One. 2024 Apr 2;19(4):e0298870. doi: 10.1371/journal.pone.0298870 (PMC10986970; doi:10.1371/journal.pone.0298870)

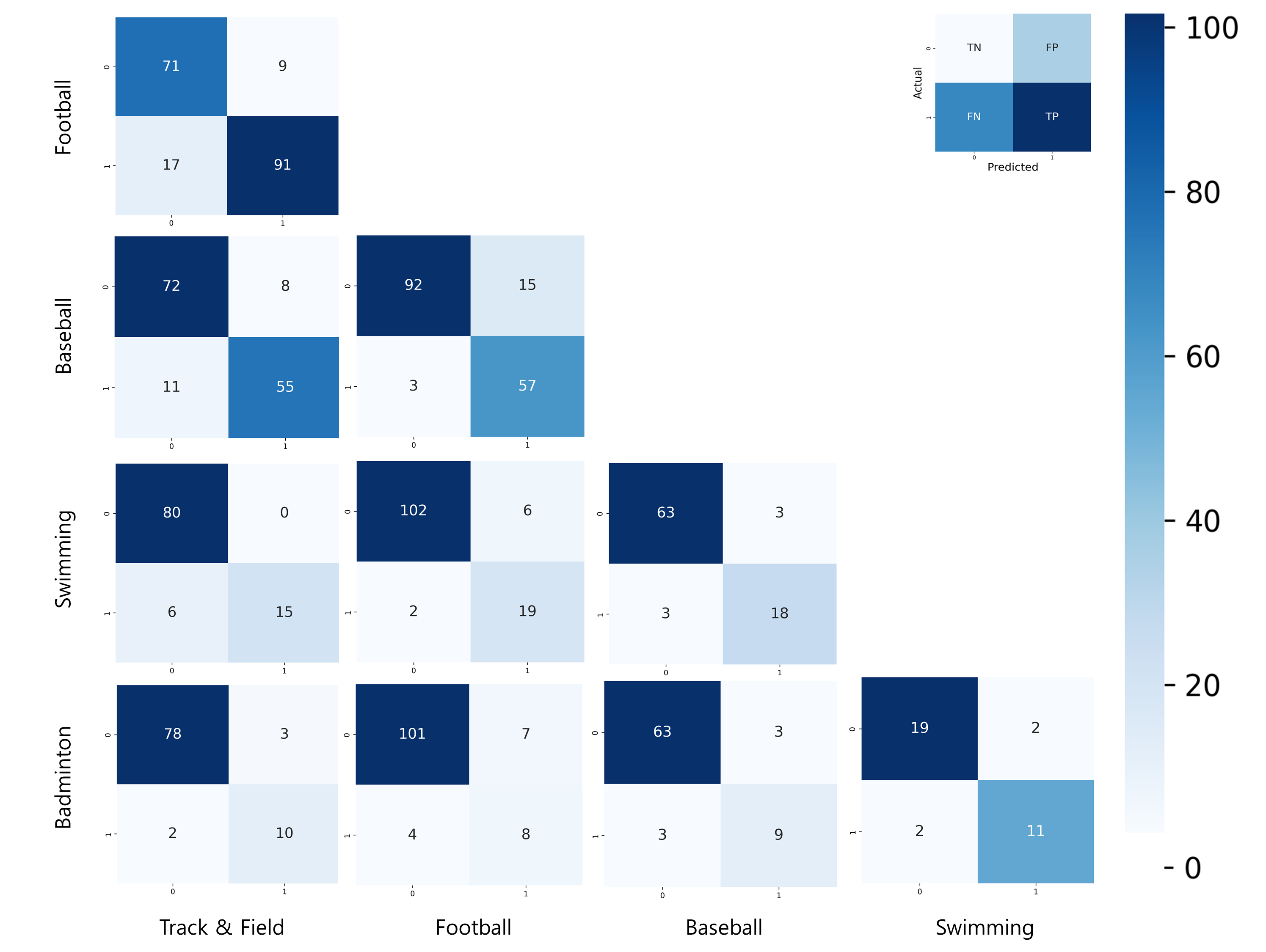

Supplement: S1 Fig — (TIF) [file pone.0298870.s002.TIF]

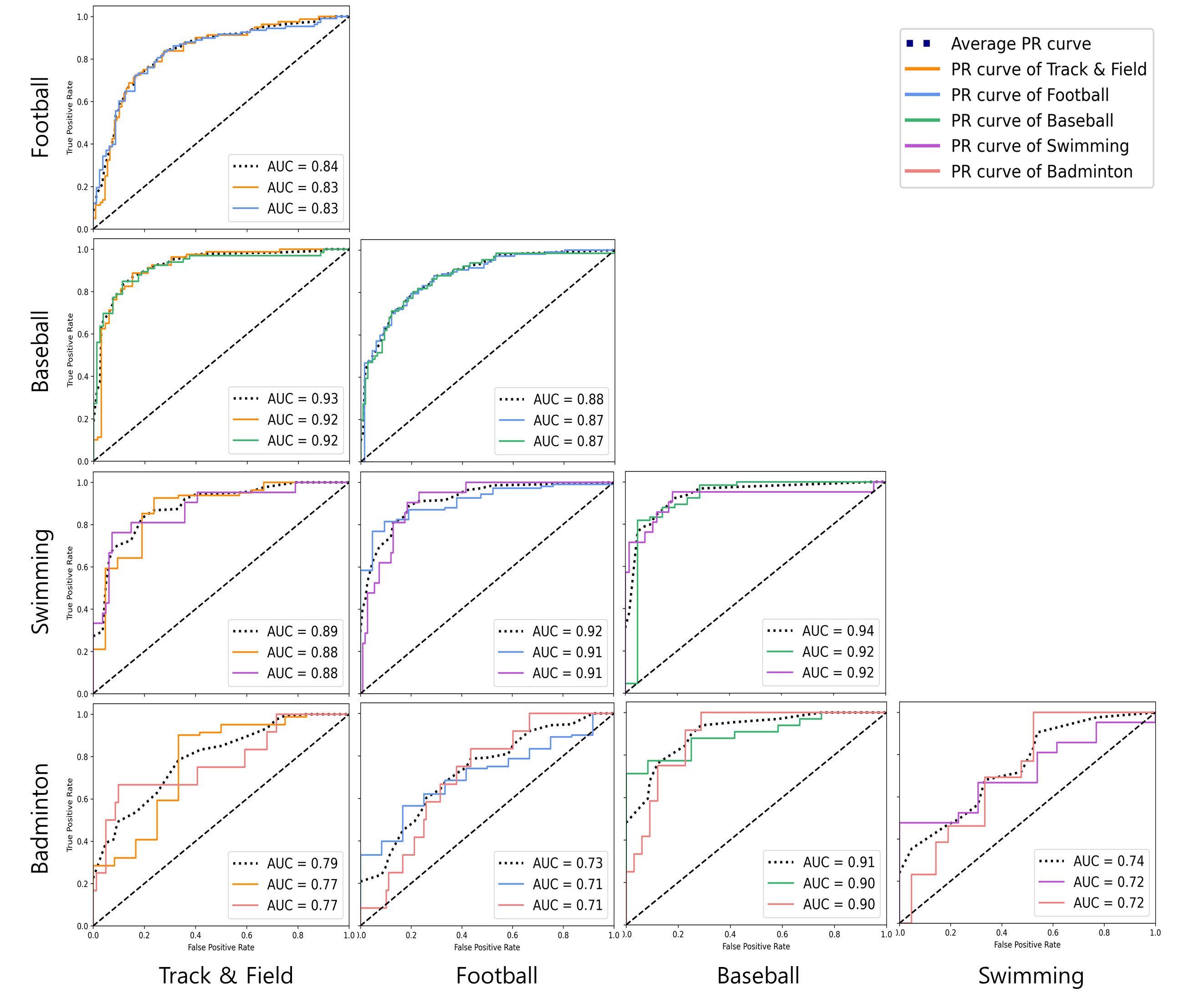

Supplement: S2 Fig — (TIF) [file pone.0298870.s003.tif]

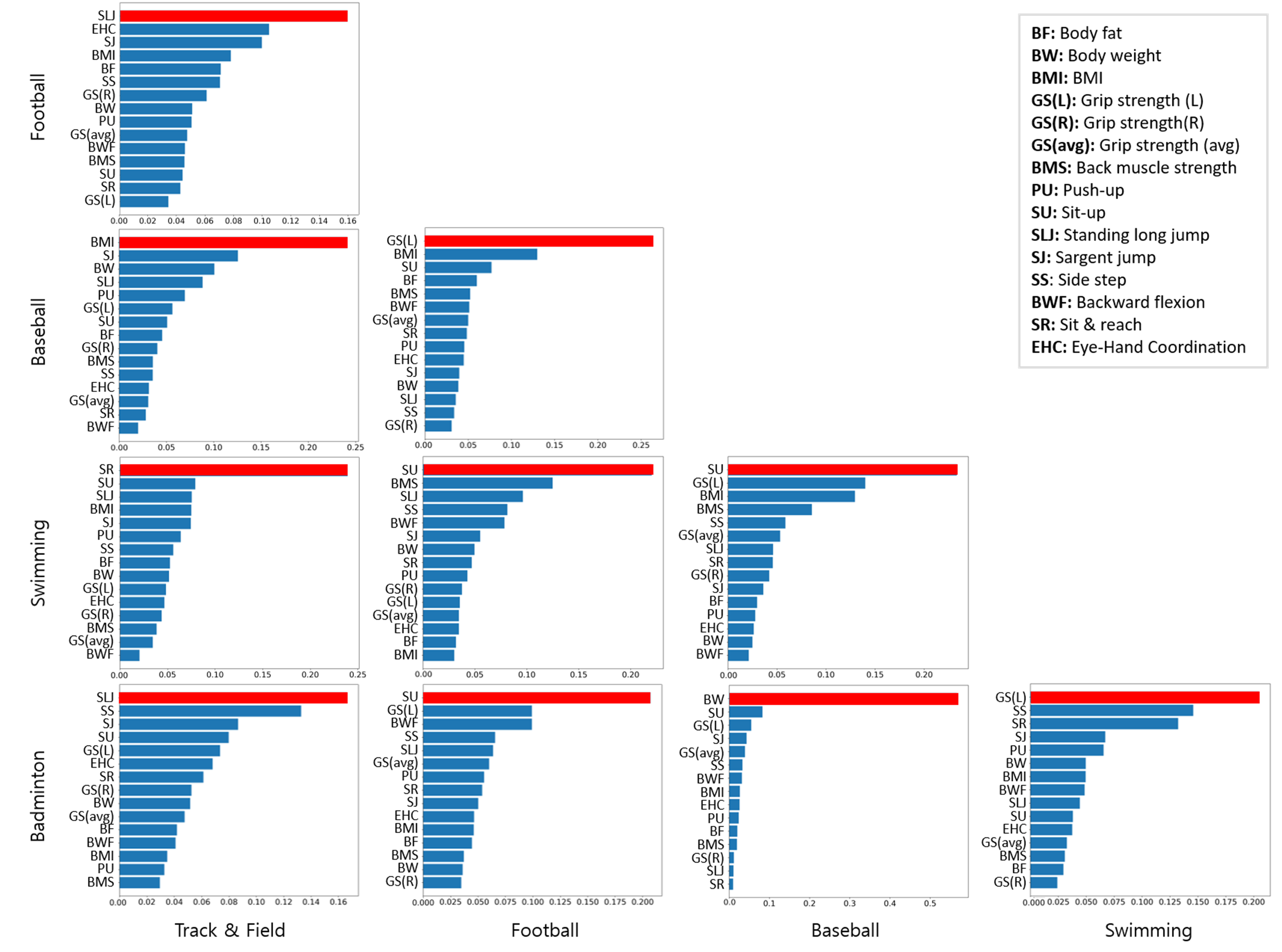

Supplement: S3 Fig — The red bar plot represents essential elements of physical fitness. (TIF) [file pone.0298870.s004.tif]
